# Liraglutide Attenuates Aortic Valve Calcification in a High-Cholesterol-Diet-Induced Experimental Calcific Aortic Valve Disease Model in Apolipoprotein E-Deficient Mice

**DOI:** 10.3390/jcdd10090386

**Published:** 2023-09-06

**Authors:** Yangzhao Zhou, Zhaoshun Yuan, Min Wang, Zhiyuan Zhang, Changming Tan, Jiaolian Yu, Yanfeng Bi, Xiaobo Liao, Xinmin Zhou, Md Sayed Ali Sheikh, Dafeng Yang

**Affiliations:** 1 Department of Cardiovascular Surgery, The Second Xiangya Hospital of Central South University, Changsha 410011, China; yangzhaochou@csu.edu.cn (Y.Z.); zsy7107@csu.edu.cn (Z.Y.); bsscwm@163.com (M.W.); zhiyuan256@sina.com (Z.Z.); tanchangming79@csu.edu.cn (C.T.); y1244373010@163.com (J.Y.); banma323517@163.com (Y.B.); baileyliaows@126.com (X.L.); xmzhou371@csu.edu.cn (X.Z.); 2 Department of Internal Medicine, Cardiology, College of Medicine, Jouf University, Sakaka 72388, Saudi Arabia; drsheikh07@hotmail.com

**Keywords:** calcific aortic valve disease, liraglutide, glucagon-like peptide-1, glucagon-like peptide-1 receptor

## Abstract

Background: Calcific aortic valve disease (CAVD) is a significant cause of morbidity and mortality among elderly people. However, no effective medications have been approved to slow or prevent the progression of CAVD. Here, we examined the effect of liraglutide on aortic valve stenosis. Methods: Male *Apoe^−/−^* mice were fed with a high-cholesterol diet for 24 weeks to generate an experimental CAVD model and randomly assigned to a liraglutide treatment group or control group. Echocardiography and immunohistological analyses were performed to examine the aortic valve function and morphology, fibrosis, and calcium deposition. Plasma Glucagon-like peptide-1 (GLP-1) levels and inflammatory contents were measured via ELISA, FACS, and immunofluorescence. RNA sequencing (RNA-seq) was used to identify liraglutide-affected pathways and processes. Results: Plasma GLP-1 levels were reduced in the CAVD model, and liraglutide treatment significantly improved aortic valve calcification and functions and attenuated inflammation. RNA-seq showed that liraglutide affects multiple myofibroblastic and osteogenic differentiations or inflammation-associated biological states or processes in the aortic valve. Those liraglutide-mediated beneficial effects were associated with increased GLP-1 receptor (GLP-1R) expression. Conclusions: Liraglutide blocks aortic valve calcification and may serve as a potential therapeutic drug for CAVD treatment.

## 1. Introduction

Calcific aortic valve disease (CAVD) is a significant cause of morbidity and mortality that affects approximately 5% of adults above the age of 65 years due to heart failure caused by hemodynamic alteration resulting from aortic valve stenosis [1]. Moreover, the prevalence of CAVD is rapidly growing due to an increasing life expectancy and aging population [2,3]. Unfortunately, no effective medications are approved to slow or prevent the progression of CAVD [4,5]. Transcatheter or surgical aortic valve replacement is the only treatment shown to improve survival for symptomatic severe CAVD patients [6,7,8]. Yet, the operative risk increases with patients’ age and left ventricular dysfunction [9]. Therefore, there is an urgent need to elucidate the pathobiology of aortic valve stenosis and identify promising therapeutic treatment to slow or prevent CAVD progression.

The valvular endothelial cell (VEC) and valvular interstitial cell (VIC) are the major cell types forming the structure and function of valve cusps. Accumulating evidence demonstrates that CAVD develops through an initial VEC dysfunction, lipid deposition and inflammation, and subsequent VIC osteogenic differentiation and calcification during disease progression [5,10,11]. Among these pathologic changes, VEC dysfunction and VIC osteogenic differentiation and calcification are hallmarks of CAVD [10]. Multiple key signaling pathways and regulatory genes contribute to VEC dysfunction and VIC calcification. For instance, NOTCH1 signaling pathway activation inhibits VIC calcification by reducing Runt-related transcription factor 2 (Runx2) and osteocalcin expression, and NOTCH1 mutations cause CAVD in both humans and mice [12,13,14]. Activated nuclear factor kappa light-chain enhancer of activated B cells (NF-kB) signaling was found throughout the CAVD process in both VEC and VIC, and the genetic deletion of NF-kB activation attenuates CAVD through the inhibition of p65-mediated inflammatory endothelial-to-mesenchymal transition (EndMT) in VEC and VIC calcification [15]. However, currently used drugs targeting specific pathways involved in CAVD pathogenesis have failed to show ameliorated aortic valve stenosis [4,5]. Therefore, it is necessary to discover a potential drug that inhibits multiple key processes during CAVD initiation and progression.

Glucagon-like peptide-1 (GLP-1), a gut-derived peptide hormone, was fist found to promote insulin release by targeting the pancreatic β cells and rapidly developed as a promising drug for the treatment of obesity and type 2 diabetes mellitus (T2DM), well-known risk factors of cardiovascular diseases [16]. Unexpectedly, GLP-1 and its analogs were subsequently identified to have various cardioprotective actions through binding its receptor GLP-1R [17]. For example, liraglutide, a well-known long-acting GLP-1 analogue, has been shown to improve endothelial function and reduce lipid burden and inflammation [18,19,20,21], which all play critical roles in the pathogenesis of CAVD. In addition to those beneficial effects, recent studies demonstrated that liraglutide inhibits the calcification of various cell types (e.g., vascular smooth muscle cell and embryonic osteoblast cell) through binding to GLP-1R and subsequently activating PI3K/Akt signaling [22,23,24], suggesting that liraglutide may also have effects on VIC calcification and could serve as a potential anti-CAVD therapeutic drug. Herein, we examined the effect of liraglutide on aortic valve stenosis using a high-cholesterol-diet-induced experimental CAVD model in apolipoprotein E-deficient (*Apoe^−/−^*) mice and found that liraglutide blocks aortic valve stenosis through attenuating aortic valve inflammation, VIC osteogenic differentiation, and calcification.

## 2. Materials and Methods

### 2.1. Animals and In Vivo Treatment

Our animal experiments follow the Guide for the Care and Use of Laboratory Animals published by the US National Institutes of Health and were approved by the Institutional Animal Care and Use Committee at Second Xiangya Hospital of Central South University (Protocol No. 2021810). Male *Apoe^−/−^* and C57BL/6 mice from 8 to 10 weeks old were purchased from Beijing Vital River Laboratory Animal Technology Co. in China. All mice were housed under a 12 h light/dark cycle in a pathogen-free animal facility with free access to food and water. Mice were kept on a standard chow diet or on a 1.25% high-cholesterol diet (HCD; D12108C, Research Diets New Brunswick, NJ, USA) for 24 weeks. *Apoe^−/−^* mice were randomly assigned to control group (*n* = 11) and liragultide administration group (*n* = 9). Mice were intraperitoneally injected with liraglutide (Victoza injection pen, 6 mg/mL from Novo Nordis, 30 μg/kg twice daily) or same dosage of saline (control group) every 12 h. Mice were weighed weekly, and glucose tolerance test (GTT) was performed at week 23 after liraglutide treatment. After 24 weeks, all mice underwent transthoracic echocardiography after anesthetization with isoflurane using a Vevo 2100 device equipped with 30 MHz linear-array transducer with a digital ultrasound system (VisualSonics Inc., Toronto, ON, Canada). Pulsed-wave Doppler analysis of three consecutive cardiac cycles across the aortic valve was performed and averaged along the parasternal long axis to obtain maximal transvalvular velocity. All echocardiographic examinations were performed by the same investigator blinded to the study design and animal treatment. Then, mice were humanely sacrificed with carbon dioxide narcosis, followed by cardiac puncture blood collection; aortic root and descending thoracoabodominal aorta were harvested. Some of the aortic roots were used for RNA isolation from the aortic valve (*n* = 3 per group), and the left roots (*n* = 6–8 per group) were embedded in optimum cutting temperature (OCT) compound or frozen at −80 °C, and the descending thoracoabodomial aortas were fixed in 4% paraformaldehyde (PFA) for further experiments.

### 2.2. Plasma Insulin, GLP-1 and Inflammatory Cytokine Level Measurement, and Lipid Profile Analysis

Plasma insulin (E-EL-M1382c, Elabscience, Wuhan, China), GLP-1 (E-EL-M3012, Elabscience), TNF-α (RK00027, ABclonal, Wuhan, China), IL-6 (RK00008, ABclonal), and IL-4 (RK00036, ABclonal) levels were determined via ELISA according to the manufacture’s protocols. Total cholesterol (A111-1-1), triglyceride (A110-1-1), low-density lipoprotein cholesterol (A113-1-1), and high-density lipoprotein cholesterol (A112-1-1) were measured using relative commercial colorimetric enzymatic assay kits from Najing Jiancheng Bioengineering, as described previously [25].

### 2.3. Spleen Single-Cell Preparation and Flow Cytometry Analysis

To determine the immune cell population in the spleen from saline- and liraglutide-treated *Apoe^−/−^* mice, spleen was ground and filtered through a 70 μm cell strainer (322350, BD Bioscience, Franklin Lakes, NJ, USA). Splenocytes were collected after depleting the red blood cells using red blood cell lysis buffer (5558999, BD Bioscience). Then, cells were washed with PBS and stained with the fixable viability dye eFluor 450 (65-0863-18, eBioscience, San Diego, CA, USA) and cell surface marker antibodies, including APC/Cy7-conjugated CD45 (1:200, B329320, Biolegand, San Diego, CA, USA), PE/Cy7-conjugated CD11b (1:200, B316177, Biolegand), APC-conjugated Ly6C (1:200, B325358, Biolegand), PE-conjugated CD4 (1:200, B334826, Biolegand), and PerCP-Cy5.5-conjugated CD8a (1:200, B313041, Biolegand) for subsequent FACS analysis.

### 2.4. Glucose Tolerance Test

*Apoe^−/−^* mice were fasted for 12 h and then intraperitoneally injected with D-glucose (1 g/kg, G7201, Sigma, Darmstadt, Germany). Blood glucose levels were measured before injection and at 15, 30, 60, 90, and 120 min after glucose injection using One Touch Ultra glucometer (Roche, Darmstadt, Germany).

### 2.5. Immunohistological Analysis

Serial cryostat sections (6 μm) from OCT-embedded aortic roots were prepared, and immunohistological analyses were performed as described previously [25]. Briefly, serial sections were fixed and permeabilized with cold acetone for 5 min and blocked in PBS containing 10% normal goat serum for 1 h at room temperature. Then, sections were stained with Runx2 (1:100, AF2593, Beyotime, Shanghai, China), Sox9 (1:100, A19710, Abclonal, Wuhan, China), osteocalcin (1:100, A14636, Abclonal), and Mac3 (1:100, A1464, Abclonal) for 1.5 h at room temperature, followed by appropriated biotin-conjugated secondary antibodies and HRP-streptavidin from Proteintech. Runx2-, Sox9-, osteocalcin-, and Mac3-positive areas were measured using computer-assisted image analysis software (Image-Pro Plus, Media Cybernetics, Version 6.0, Bethesda, Rockville, MD, USA) and presented as positive area per μm^2^ of aortic valve area. Masson staining kit (G1346, Solarbio, Beijing, China), Von Kossa kit (G3282, Solarbio), and Alizarin red kit (C0138, Beyotime, Shanghai, China) were used to visualize collagen and calcification deposition.

Immunofluorescent staining was performed as described previously [25]. Briefly, sections were fixed and permeabilized with cold acetone for 5 min and blocked in PBS containing 10% normal goat serum for 1 h at room temperature. Then, sections were stained with GLP-1R (1:100, A8547, Abclonal), Vimentin (1:100, AF0318, Beyotime), NF-κB p65 (1:500, ab16502, Abcam, Cambridge, England), VCAM-1 (1:250, ab134047, Abcam), NOTCH1 (1:100, A16673, Abconal), FITC-labelled anti-actin, α-smooth muscle (α-SMA, 1:500, F3777, Sigma, Darmstadt, Germany), and CD31 (1:200, ab23864, Abcam) for 3 h at room temperature, followed by incubation with Alexa Fluor 555 (1:300, A21434, Invitrogen, Waltham, MA, USA) or 488 (1:300, A11034, Invitrogen)-labelled secondary antibody for 1 h at room temperature. DAPI (P36935, Invitrogen) was used to stain the nuclei. All images were captured using a Nikon A1 confocal laser scanning microscope (Nikon, Tokyo, Japan). GLP-1R-, VCAM-1-, and NOTCH1-positive areas were measured using Image-Pro Plus software and presented as positive area per μm^2^ of the aortic valve. p65 accumulation in nucleus was measured using Image-Pro Plus software and presented as mean intensity of nucleus in the aortic valve.

Oil red O (1320-06-5, Sigma) staining was used to determine atherosclerotic lesion size in aortic root and the descending thoracoabodominal aortas.

### 2.6. RNA Isolation from Aortic Valve and Peripheral Blood Mononuclear Cells (PBMCs) and Real-Time qPCR and RNA-Seq Analysis

Aortic valve tissues were carefully isolated under a microscope (Olympus, Tokyo, Japan). PBMCs were isolated using a monocyte lymphocyte separation medium (P4350, Solarbio). TRIzol reagent (15596018, Invitrogen) was used for total RNA isolation from the aortic valve and PBMCs. PrimeScript RT reagent kit (RR047A, Takara, Osaka, Japan) was used to generate cDNA, and TB Green Premix EX Tag kit (RR820A, Takara) was used for real-time qPCR with the real-time PCR system (Roche) following the manufacturer’s instruction. Primers are listed in Table S1.

RNA-Seq analysis was performed as previously described [26]. Briefly, standard library for RNA-Seq analysis was constructed using Illumina Novaseq platform (Novogene). After quality examination using FastQC method, raw reads were used to generate the library and for subsequent sequencing. Clean reads were aligned to UCSC build mm10 of the mouse genome and augmented with transcript information from Ensembl release 79 using Hisat2 v2.0.5. DESeq2 was used to analyze the gene differential expression. Genes with adjusted *p* value < 0.05 and log2fold-change (>1.5) were called differentially expressed genes for each comparison. Gene set enrichment analysis (GSEA) was used to identify the biological states and processes [27].

### 2.7. Statistical Analyses

GraphPad 8.0 software package (GraphPad Software, Inc., La Jolla, CA, USA) was used for statistical analysis. Unpaired two-tailed Student’s *t* test was used to determine statistical significance between two groups for normally distributed continuous variables. For data without normal distribution, the non-parametric Mann–Whitney *U* test or Kruskal–Wallis test was used. All data throughout the paper are expressed as means ± SEM. *p* < 0.05 was considered significant for all tests.

## 3. Results

### 3.1. Liraglutide Treatment Ameliorates Aortic Valve Calcification in a High-Cholesterol-Diet-Fed Apoe^−/−^ Mice Model

**Figure 1:**
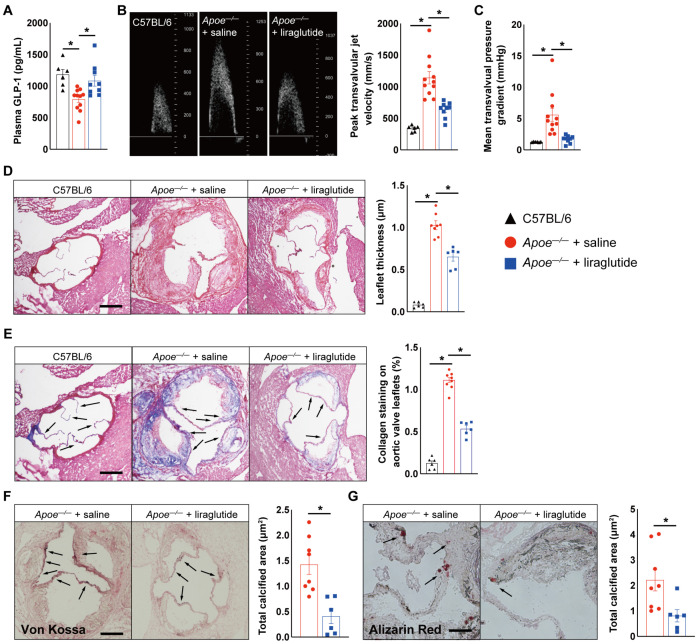
Liraglutide treatment blocks aortic valve calcification in vivo. (**A**) Plasma GLP-1 levels in C57BL/6 mice fed with normal chow diet and HCD-fed *Apoe^−/−^* mice treatment with saline or liraglutide for 24 weeks (*n* = 6–11 mice per group). Echocardiographic data in saline- or liraglutide-treated *Apoe^−/−^* mice or age-matched C57BL/6 mice fed with normal chow diet (*n* = 6–11 mice per group). (**B**) Peak transvalvular jet velocity, and (**C**) mean transvalvular pressure gradient. (**D**) Hematoxylin and eosin staining of aortic valve leaflets from saline- or liraglutide-treated *Apoe^−/−^* mice or age-matched C57BL/6 mice fed with normal chow diet (*n* = 6–8 mice per group). Scale: 200 μm. (**E**) Masson’s trichrome staining of aortic valve leaflets from saline- or liraglutide-treated *Apoe^−/−^* mice or age-matched C57BL/6 mice fed with normal chow diet. Black arrow indicates collagen deposition area (*n* = 6–8 mice per group). Scale: 200 μm. (**F**) Von Kossa staining of aortic valve leaflets from saline- or liraglutide-treated *Apoe^−/−^* mice. Black arrow indicates calcification deposition area (*n* = 6–8 mice per group). Scale: 200 μm. (**G**) Alizarin Red staining of aortic valve leaflets from saline- or liraglutide-treated *Apoe^−/−^* mice. Black arrow indicates calcification deposition area (*n* = 6–8 mice per group). Scale: 100 μm. Data shown are mean ± SEM. * *p* < 0.05.

It is well known that GLP-1 actions are transduced by GLP-1R. Indeed, multiple cells express GLP-1R, e.g., pancreatic exocrine cells, endothelial cells (ECs), vascular smooth muscle cells (VSMCs), neurons, and others, suggesting that GLP-1 targets a broad spectrum of cells and exhibits multiple functions, except its action on islet cells to potentiate glucose-dependent insulin secretion [16,28]. To examine whether aortic valve cells also express GLP-1R, we first performed immunofluorescent double staining with antibodies against GLP-1R and CD31 or GLP-1R and vimentin in aortic root sections from normal chow-diet-fed wild-type C57BL/6 mice to identify VECs and VICs, the main cell populations of the aortic valve. We found that both VECs and VICs expressed GLP-1R (Figure S1). Moreover, the expression of GLP-1R on aortic valve cells was higher than other cell types in the vasculature and heart, e.g., cardiomyocytes and VSMCs (Figure S1). Furthermore, the levels of circulating GLP-1 were reduced by 28.6% in HCD-fed *Apoe^−/−^* mice compared to C57BL/6-fed control mice with a normal chow diet but increased in *Apoe^−/−^* mice treatment with liraglutide (Figure 1A). These data suggest that the GLP-1/GLP-1R axis may play a role in VEC and VIC functions, and the dysregulation and/or inactivation may contribute to the pathogenesis of CAVD.

**Figure 2:**
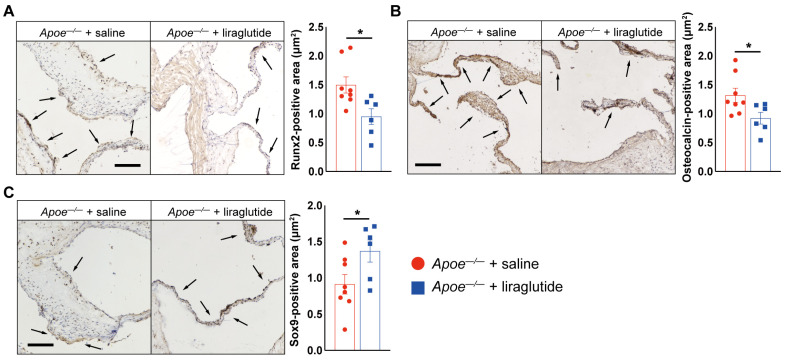
Liraglutide treatment reduces osteogenic differentiation gene expression in aortic valves of *Apoe^−/−^* mice. (**A**) Representative images and quantification show Runx2 staining in aortic valve of saline- or liraglutide-treated *Apoe^−/−^* mice. Black arrow indicates Runx2-positive area in aortic valves (*n* = 6–8 mice per group). Scale: 100 μm. (**B**) Representative images and quantification show osteocalcin staining in aortic valve of saline- or liraglutide-treated *Apoe^−/−^* mice. Black arrow indicates osteocalcin-positive area in aortic valves (*n* = 6–8 mice per group). Scale: 100 μm. (**C**) Representative images and quantification show Sox9 staining in aortic valve of saline- or liraglutide-treated *Apoe^−/−^* mice. Black arrow indicates Sox9-positive area in aortic valves (*n* = 6–8 mice per group). Scale: 100 μm. Data shown are mean ± SEM. * *p* < 0.05.

To interrogate whether liraglutide improves aortic valve calcification, *Apoe^−/−^* mice were fed with a high-cholesterol diet (HCD) and treated daily with liraglutide or saline every 12 h. After 24 weeks of HCD feeding, we observed that saline-treated *Apoe^−/−^* mice exhibited a 236% increase in peak transvalvular jet velocity (Figure 1B) and a 344% increase in mean transvalvular pressure gradient (Figure 1C) compared to age-matched C57BL/6 mice fed with normal chow diet, as measured via echocardiography, while liraglutide-treated *Apoe^−/−^* mice exhibited a 44% reduction in peak transvalvular jet velocity (Figure 1B) and a 69.8% reduction in mean transvalvular pressure gradient (Figure 1C) compared to saline-treated *Apoe^−/−^* mice, suggesting a protective role of liraglutide in HCD-induced aortic valve calcification in *Apoe^−/−^* mice. Moreover, these findings were further supported by histological analysis, evidence of aortic valve leaflet morphology, fibrosis, and calcium deposition detected through HE, Masson, Von Kossa, and Alizarin red staining, showing that liraglutide treatment significantly reduced aortic valve leaflet thickness and collagen and calcium deposition (Figure 1D–G). Furthermore, immunohistochemistry staining showed that Runx2 and osteocalcin expression in aortic valve leaflet sections of liraglutide-treated *Apoe^−/−^* mice was reduced by 36.5% and 30.1%, respectively, whereas Sox9 expression was increased by 150%, compared to those *Apoe^−/−^* mice treated with saline, indicating that osteogenesis was inhibited by liraglutide treatment (Figure 2A–C). Finally, we also observed a significantly lower body-weight gain and improved glucose tolerance, as well as reduced atherosclerotic lesion and attenuated lipid metabolism, in liraglutide-administrated *Apoe^−/−^* mice (Figures S2 and S3). However, the administration of liraglutide did not alter plasma insulin and random blood glucose levels (Figure S2). Taken together, these results suggest that liraglutide treatment leads to a potent attenuation of aortic valve calcification.

### 3.2. Liraglutide Treatment Inhibits Osteogenic Differentiation and Inflammation-Associated Singling Pathway

**Figure 3:**
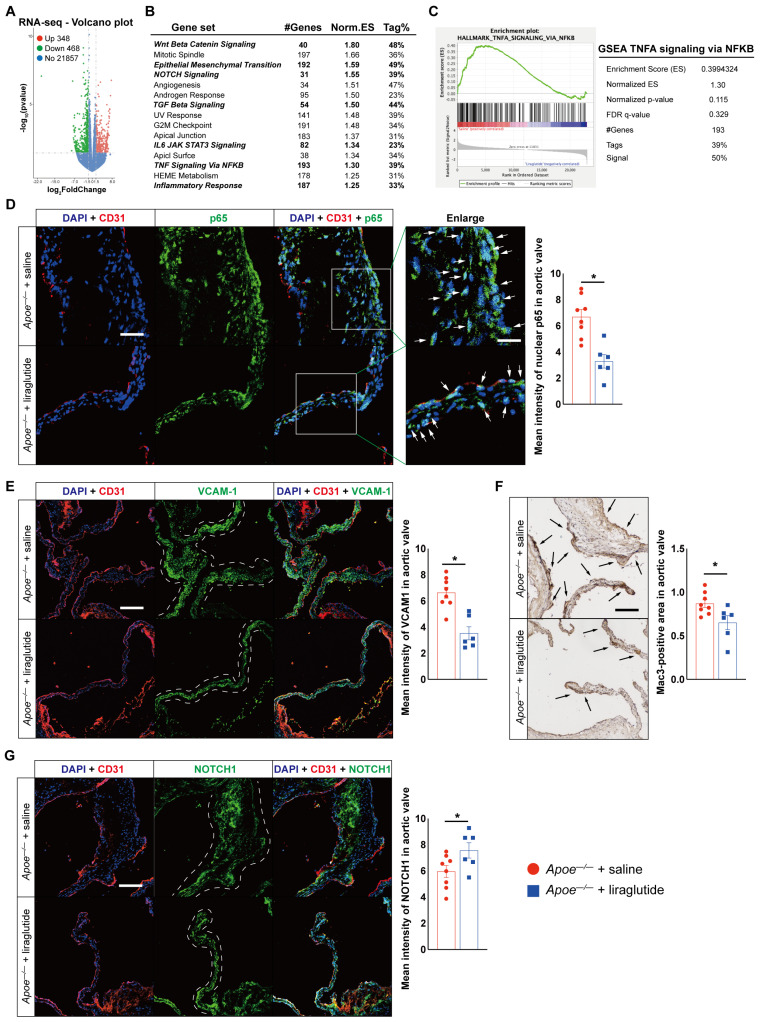
Downstream consequence of liraglutide treatment on aortic valves in HCD-induced experimental CAVD in *Apoe^−/−^* mice. (**A**) RNA-seq profiling of aortic valves from saline- or liraglutide-treated *Apoe^−/−^* mice. Volcano plot displaying significantly dysregulated genes (*p* Value < 0.05; log_2_ fold change > 1.5) (*n* = 3 mice per group). (**B**) GSEA of the top 15 significantly affected processes and (**C**) enrichment plot for the TNF signaling via NFKB of (**A**) after liraglutide treatment. Representative images and quantification show p65 accumulation in nuclear (**D**), VCAM−1 expression (**E**) in aortic valves from saline- or liraglutide-treated *Apoe^−/−^* mice (*n* = 6–8 mice per group). Frozen sections of aortic sinus were stained for anti-p65 or VCAM-1 (green), anti-CD31 (red), and DAPI (blue). The dashed-line area indicates aortic valves. Arrows indicates accumulated p65 in nuclear. Scale: 50 μm. Enlarged scale 25 μm. (**F**) Representative images and quantification show Mac3-positive area in aortic valve from saline- or liraglutide-treated *Apoe^−/−^* mice (*n* = 6–8 mice per group). Black arrow indicates Mac3-positive area in aortic valves. Scale: 100 μm. (**G**) Representative images and quantification show NOTCH1 expression in aortic valves from saline- or liraglutide-treated *Apoe^−/−^* mice (*n* = 6–8 mice per group). Frozen sections of aortic sinus were stained for NOTCH1 (green), anti-CD31 (red), and DAPI (blue). The dashed-line area indicates aortic valves. Scale: 50 μm. Data shown are mean ± SEM. * *p* < 0.05.

We next sought to explore the mechanisms by which liraglutide treatment ameliorates aortic valve calcification. To identify the highly regulated pathways and processes affected by liraglutide treatment, we performed RNA-seq using RNA isolated from the aortic valve of HCD-fed *Apoe^−/−^* mice treated with saline or liraglutide. We identified a number of up- or down-regulated genes (Figure 3A). Moreover, using GSEA analysis [27], we found various myofibroblastic and osteogenic differentiations or inflammation-associated biological states or processes among the top 15 hallmark gene sets, including WNT BETA CATENIN signaling, NOTCH signaling, TGF Beta signaling, IL6 JAK STAT3 signaling, TNFA signaling via NFKB, and inflammatory response (Figure 3B,C). Consistent with these observations, the accumulation of NF-κB p65 in nuclei, expression of vascular cell adhesion molecule-1 (VCAM-1), and Mac3-positive macrophage content within the aortic valve were reduced by 50.8%, 46.8%, and 25.1%, respectively, in *Apoe^−/−^* mice treated with liraglutide (Figure 3D–F), whereas the expression of NOTCH1, a critical protein in controlling aortic valve calcification and CAVD in both mice and humans [12,13], was increased by 127% in liraglutide-treated *Apoe^−/−^* mice compared to saline-treated *Apoe^−/−^* mice (Figure 3G).

**Figure 4:**
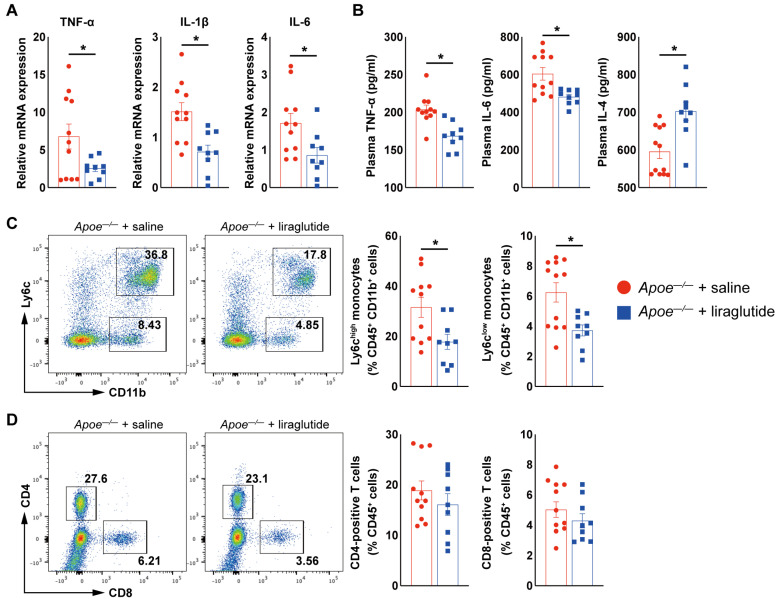
Liraglutide treatment inhibits inflammation in HCD-induced experimental CAVD model in *Apoe^−/−^* mice. (**A**) Real-time qPCR analysis of TNF−α, IL−1β, and IL−6 in PBMCs from saline- or liraglutide-treated *Apoe^−/−^* mice (*n* = 9–11 mice per group). The expression of genes was normalized to mouse β-actin. (**B**) ELISA analysis of TNF−α, IL−6, and IL−4 plasma from aortic valve of saline- or liraglutide-treated *Apoe^−/−^* mice (*n* = 9–11 mice per group). (**C**) FACS analysis of splenic Ly6c^high^ and Ly6c^low^ monocytes form saline- or liraglutide-treated *Apoe^−/−^* mice (*n* = 9–11 mice per group). (**D**) FACS analysis of splenic CD4-positive and CD8-positive T cells from saline- or liraglutide-treated *Apoe^−/−^* mice (*n* = 9–11 mice per group). Data shown are mean ± SEM. * *p* < 0.05.

In addition to local inflammatory responses in the aortic valve, systemic inflammation also contributes importantly to the pathogenesis of CAVD [5,10,11]. In line with the findings in the aortic valve (Figure 1, Figure 2 and Figure 3), we found that liraglutide treatment reduced the expression of TNFα, IL-1β, and IL-6 by 62.3%, 53%, and 48.2%, respectively, in peripheral blood mononuclear cells (PBMCs) (Figure 4A). Moreover, circulating pro-inflammatory cytokines, including TNFα and IL-6, in the plasma were also reduced, whereas increased plasma IL-4 levels were observed in *Apoe^−/−^* mice treated with liraglutide (Figure 4B). Furthermore, FACS showed that the percentages of splenic Ly6C^high^ and Ly6C^low^ monocytes were reduced in liraglutide-treated *ApoE^−/−^* mice, with a trend to reduce CD4^+^ and CD8^+^ T-cell contents in the spleen (Figure 4C,D). Together, these findings (Figure 3 and Figure 4) indicate that liraglutide inhibits VIC osteogenic differentiation and inflammation and, in turn, attenuates aortic valve calcification.

### 3.3. Liraglutide Treatment Increases GLP-1R Expression

**Figure 5:**
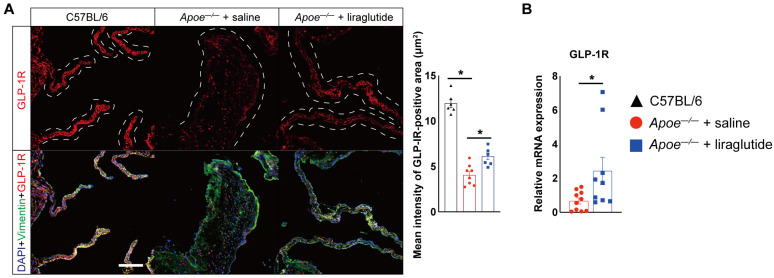
Liraglutide treatment increases GLP-1R expression in HCD-induced experimental CAVD model in *Apoe^−/−^* mice. (**A**) Representative images and quantification show GLP-1R expression in aortic valves from saline- or liraglutide-treated *Apoe^−/−^* mice or age-matched C57BL/6 mice fed with normal chow diet (*n* = 6–8 mice per group). Frozen sections of aortic sinus were stained for anti-GLP-1R, (red), anti-vimentin (green), and DAPI (blue). The dashed-line area indicates aortic valves. Scale: 50 μm. (**B**) Real-time qPCR analysis of GLP-1R expression in PBMCs from saline- or liraglutide-treated *Apoe^−/−^* mice (*n* = 9–11 mice per group). The expression of genes was normalized to mouse β-actin. Data shown are mean ± SEM. * *p* < 0.05.

Liraglutide is a synthetic GLP-1 analogue, and the actions of GLP-1 and its analogues are dependent on GLP-1R expression. Multiple cells express GLP-1R [16,28]. We also identified that GLP-1R was expressed in aortic VICs and VECs (Figure S1). A previous study demonstrated that liraglutide increased vascular endothelial GLP-1R expression, and its expression contributed importantly to liraglutide-mediated cardiovascular protection in mice with experimental arterial hypertension [17]. Thus, we asked whether liraglutide also increases GLP-1R expression in VICs and VECs. As shown in Figure 5A, the expression of GLP-1R was decreased by 70% in the aortic valve of saline-treated *Apoe^−/−^* mice compared to age-matched C57BL/6 mice fed with normal chow diet but increased by 149% in the aortic valve in response to liraglutide treatment compared to *Apoe^−/−^* mice treated with saline. Moreover, in line with reduced inflammatory cytokine expression in PBMCs (Figure 4A), we also observed that the GLP-1R expression was significantly increased by 3.64-fold in PBMCs from liraglutide-treated *Apoe^−/−^* mice compared with those from *Apoe^−/−^* mice treated with saline (Figure 5B). Collectively, these results indicated that liraglutide increases GLP-1R expression, and a positive feedback loop between GLP-1 and GLP-1R may be involved in liraglutide-mediated cardiovascular protective effects.

## 4. Discussion

Here, we found that the plasma GLP-1 levels were reduced, and liraglutide, a long-acting GLP-1 analog, inhibited osteogenic differentiation and inflammation, reducing the aortic valve leaflet thickness and collagen and calcium deposition, an effect eventually resulting in attenuated aortic valve stenosis in an HCD-induced experimental CAVD model in *Apoe^−/−^* mice. Moreover, we observed that liraglutide treatment slowed down body-weight gain, improved glucose tolerance, reduced lipid and atherosclerotic burden, but had no effect on glycemia and insulin levels. Furthermore, these liraglutide-mediated beneficial effects were associated with increased GLP-1R expression. Taken together, these findings pinpoint a previously unidentified role of the GLP-1/GLP-1R axis in the pathogenesis of CAVD and indicate that liraglutide may serve as a potential therapeutic drug for preventing aortic valve calcification.

Evidence from clinical and experimental studies demonstrated a potent protective role of GLP-1 analogues in patients with cardiovascular disease (CVD) [29,30,31,32,33]. For example, the LEADER trial found that liraglutide treatment decreased the incidence of major cardiovascular events, cardiovascular death, and all-cause death by 13%, 22%, and 15%, respectively, in T2DM patients with CVD compared to those patients treated with placebo [29]. Moreover, liraglutide also reduced the myocardial infarction (MI) incidence in high-risk T2DM patients, as well as improving the clinical outcomes of MI [30]. However, whether liraglutide has similar beneficial effects on CAVD remains unclear. We examined the effect of liraglutide on aortic valve calcification in an HCD-induced *Apoe^−/−^* mice model. In concordance with previous studies performed by other groups, we showed that liraglutide exhibited multiple cardiovascular protective effects, including improved glucose and lipid metabolism, attenuated endothelial dysfunction, and reduced inflammation and atherosclerotic burden. Moreover, liraglutide treatment significantly ameliorated aortic valve stenosis and calcification, as indicated through echocardiography and histopathological examination. CAVD is a highly complex and multifactorial disease (e.g., elevated LDL cholesterol, metabolic syndrome, inflammation, and so on), and, currently, no effective pharmacotherapy is available to prevent CAVD progression [10,34,35]. Indeed, multiple clinical trials informed us that lipid-lowering therapy [36,37,38,39] or renin–angiotensin system inhibition [40,41,42,43] did not reduce aortic valve calcification or improve clinical outcomes. Collectively, our results suggest that liraglutide, unlike statins that specifically target the lipid metabolism, inhibits multiple key risk factors that promote aortic valve calcification and may serve as a promising therapeutic treatment for preventing CAVD.

GLP-1 was first found to exert incretin-like activity, promoting glucose-dependent insulin secretion in normal and diabetic animals and humans through binding GLP-1R [28,44]. However, we did not observe an insulin alteration in those *Apoe^−/−^* mice. Indeed, accumulating evidence demonstrated that, except pancreatic β cells, multiple cell types express GLP-1R, suggesting that GLP-1 exhibits various functions rather than simple potentiate glucose-dependent insulin secretion, and a cell-type-selective GLP-1R activation manner was required for GLP-1 or GLP-1 analogues. For example, GLP-1R agonists improve outcomes in ischemic heart diseases; however, this beneficial effect does not require cardiomyocyte GLP-1R [45]. In an ATII-induced experimental hypertension mice model, GLP-1R activation in endothelial cells by liraglutide inhibited leukocyte vessel wall infiltration, resulting in lowered oxidative stress and reduced endothelial NO synthase (eNOS) uncoupling, which eventually prevented vascular remodeling [17]. Moreover, these liraglutide-mediated cardiovascular protections did not require GLP-1R on myeloid cells [17]. Furthermore, liraglutide improves endothelial dysfunction and inhibits inflammation, thereby attenuating atherosclerosis [18,19,20,21]. In the present study, we found that circulating GLP-1 levels were lower in HCD-fed *Apoe^−/−^* mice compared to C57BL/6 mice fed with normal chow diet but increased in *Apoe^−/−^* mice treated with liraglutide. This result was consistent with a recent study demonstrating that GLP-1 levels were reduced in calcific aortic valves and serum in CAVD patients [46]. Moreover, we identified that GLP-1R was also expressed on VECs and VICs, suggesting a potential role for the GLP-1/GLP-1R axis in CAVD. Furthermore, the liraglutide-mediated improvement in aortic valve fibrosis and calcification and cardiovascular protective effects was associated with elevated GLP-1R expression and improved VEC function (decreased NF-κB p65 accumulation in nuclei and VCAM-1 expression), indicating a potential positive feedback loop between liraglutide and GLP-1R expression and, in turn, facilitating GLP-1/GLP-1R axis-mediated beneficial effects. Our RNA-seq and immunohistochemical data that liraglutide treatment affected multiple pathways involved in inflammation (e.g., inflammatory response, TNFA signaling via NFKB, and IL6 JAK STAT3 signaling) and calcification (e.g., NOTCH signaling, TGF Beta signaling, and WNT BETA CATENIN signaling) further supported this hypothesis. In fact, VEC dysfunction, inflammation, and lipid disposition play critical roles in the initiation of CAVD, whereas osteogenic differentiation and calcification contribute importantly to disease progression [5,10]. Collectively, these results suggest that the pathogenesis of CAVD is complicated and involves a larger gene network modulation [11,47], and liraglutie may serve as switch of several critical pathways, controlling osteogenic differentiation and inflammation through activating GLP-1R.

There are several limitations to our study. Frist, growing evidence suggests that a cell-type-selective GLP-1R activation manner plays critical role in GLP-1R agonist-mediated cardiovascular protection, and endothelial GLP-1R may be the predominant one. We cannot rule out other cell types that contribute to the beneficial effects of liraglutide in the context of CAVD. Future work needs to clarify this question using a cell-type- or tissue-specific GLP-1R-deficient transgenic mice model. Second, native GLP-1 can be cleaved by dipeptidyl peptidase 4 (DPP-4) to generate GLP-1(9-36)amide, which, in turn, mediates the cardioprotective actions of GLP-1 through a GLP-1R-independent pathway [48,49]. Thus, identifying GLP-1R-independent mechanisms will be critical to fully elucidate how GLP-1 ameliorates aortic valve calcification.

## 5. Conclusions

Given that several randomized clinical trials that are testing drugs targeting specific pathways or targets involved in CAVD pathogenesis are still ongoing [5], our findings may have clinical implications, as they suggest that liraglutide blocks aortic valve calcification formation and may be a potential therapeutic drug for the treatment of CAVD.

## Data Availability

All data relevant to the study are included in the article. The data are available from the corresponding authors on reasonable request.

## Supplementary Materials

The following supporting information can be downloaded at: https://www.mdpi.com/article/10.3390/jcdd10090386/s1, Figure S1: GLP-1R expresses on aortic valvular endothelial and interstitial cells; Figure S2: Liraglutide treatment inhibits body weight gain and improves glucose tolerance, but not alter random blood glucose and plasma insulin levels; Figure S3: Liraglutide treatment inhibits atherosclerotic lesion formation and improve lipid metabolism; Table S1: Primer list.
