# Supplementary material for: Liraglutide Attenuates Aortic Valve Calcification in a High-Cholesterol-Diet-Induced Experimental Calcific Aortic Valve Disease Model in Apolipoprotein E-Deficient Mice
Source: J Cardiovasc Dev Dis. 2023 Sep 6;10(9):386. doi: 10.3390/jcdd10090386 (PMC10531705; doi:10.3390/jcdd10090386)
Supplement: Supplementary file 1 [file jcdd-10-00386-s001.zip › jcdd-2545027-supplementary.pdf]

Supplementary Information

Liraglutide Attenuates Aortic Valve Calcification in A High Cholesterol Diet-induced Experimental Calcific Aortic Valve Disease Model in Apolipoprotein E-Deficient Mice

Yangzhao Zhou[1], Zhaoshun Yuan[1], Min Wang[1], Zhiyuan Zhang[1], Changming Tan[1], Jiaolian Yu[1], Yanfeng Bi[1], Xiaobo Liao[1], Xinmin Zhou[1], Md Sayed Ali Sheikh[2], Dafeng Yang[1]

[1]Department of Cardiovascular Surgery, The Second Xiangya Hospital of Central South University, Changsha, Hunan 410011, China

[2]Department of Internal Medicine, Cardiology, College of Medicine, Jouf University, Sakaka, Saudi Arabia

Corresponding author: Dafeng Yang, MD, PhD, Department of Cardiovascular Surgery, The Second Xiangya Hospital, Central South University, 139 Middle Renming Road, Changsha, Hunan 410011, China. Email: dafengyang@csu.edu.cn.

Supplementary Figure S1. GLP-1R expresses on aortic valvular endothelial and interstitial cells.

Supplementary Figure S2. Liraglutide treatment inhibits body weight gain and improves glucose tolerance, but not alter random blood glucose and plasma insulin levels.

Supplementary Figure S3. Liraglutide treatment inhibits atherosclerotic lesion formation and improves lipid metabolism.

Supplementary Table S1. Primer list.

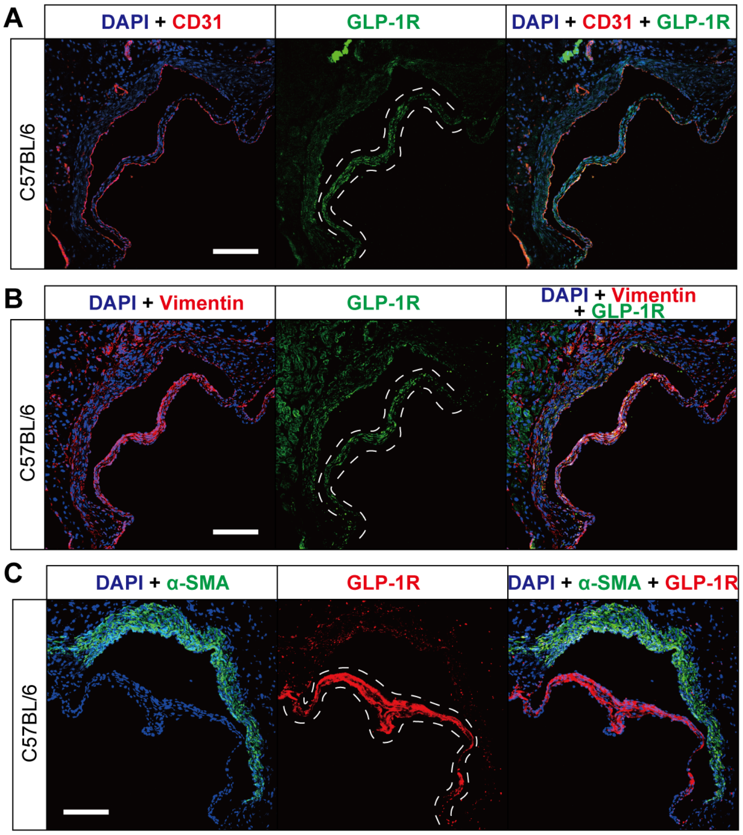

Supplementary Figure S1. GLP-1R expresses on aortic valvular endothelial and interstitial cells. Frozen sections of aortic sinus were stained for anti-α-SMA or GLP-1R (green), anti-vimentin, anti-CD31or anti-GLP-1R (red), and DAPI (blue). **A)** Representative images show GLP-1R expresses on CD31-positive aortic valvular endothelial cells. The dashed line area indicates aortic valves. Scale: 50 μm. **B)** Representative images show GLP-1R expresses on vimentin-positive aortic valvular interstitial cells. The dashed line area indicates aortic valves. Scale: 50 μm. **C)** Representative images show GLP-1R expresses on α-smooth muscle actin (α-SMA)-positive vascular smooth muscle cells. The dashed line area indicates aortic valves. Scale: 50 μm.

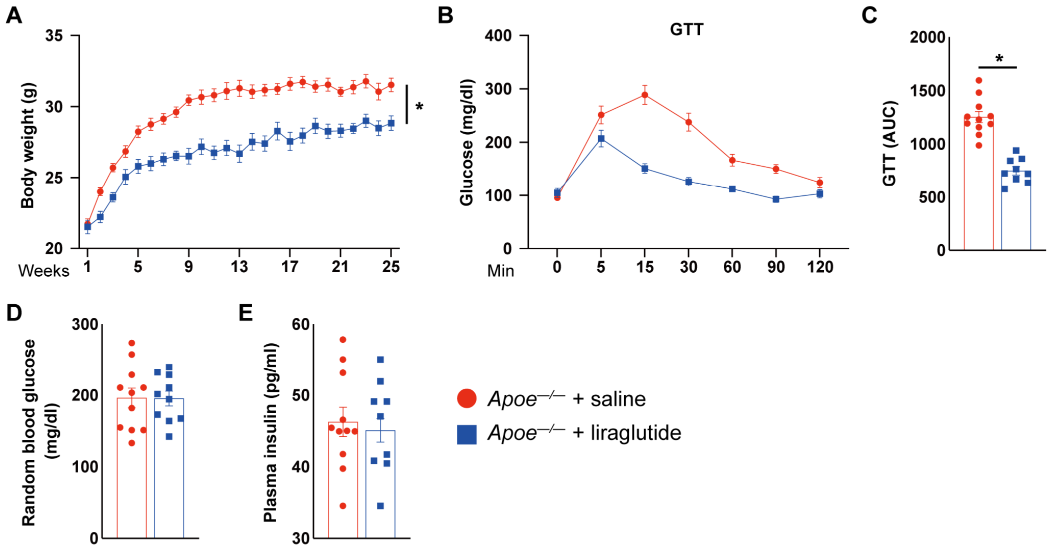

Supplementary Figure S2. Liraglutide treatment inhibits body weight gain and improves glucose tolerance, but not alter random blood glucose and plasma insulin levels. A) Body weights over time of *Apoe*$^{-/-}$ mice on HCD diet treated with saline or liraglutide. **B)** Blood glucose levels were measured on week 23 for glucose tolerance test (GTT) in *Apoe*$^{-/-}$ mice on HCD diet treated with saline or liraglutide. **C)** Area under the curve (AUC) of glucose tolerance test was quantified. **D)** Random blood glucose levels were measured on week 24. **E)** ELISA analysis of plasma insulin of *Apoe*$^{-/-}$ mice on HCD diet treated with saline or liraglutide. Data shown are mean ± SEM. *$P < 0.05$.

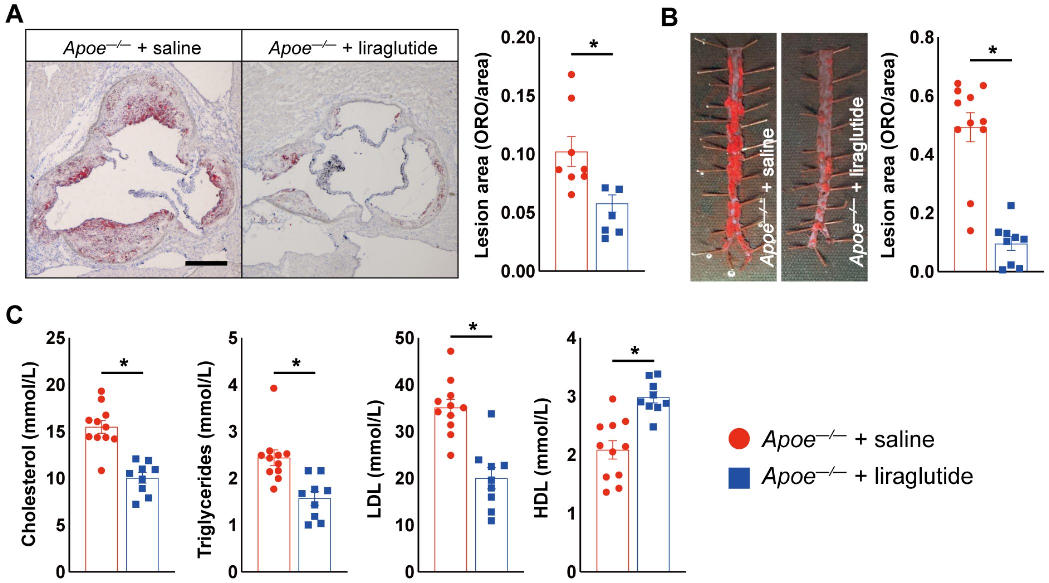

Supplementary Figure S3. Liraglutide treatment inhibits atherosclerotic lesion formation and improves lipid metabolism. A) Lesion areas were quantified by Oil Red O (ORO)-stained aortic sinus sections of *Apoe*−/− mice on HCD diet treated with saline or liraglutide. Scale: 200 μm. **B)** Lesion areas were quantified by Oil Red O (ORO)-stained thoracoabdominal aorta of *Apoe*−/− mice on HCD diet treated with saline or liraglutide. **C)** Circulating lipid levels (total cholesterol, triglycerides, LDL-C and HDL-C) in *Apoe*−/− mice on HCD diet treated with saline or liraglutide. Data shown are mean ± SEM. *$P < 0.05$.

Supplementary Table S1. Primer list.

Gene name	Forward	Reverse
Mouse-GLP-1R	CCTGTGTCCTTCACCTTCCCTA	GTACCACGGTGTCCCTCTCA
Mouse-TNF-α	CCCTCACACTCAGATCATCTTCT	GCTACGACGTGGGCTACAG
Mouse-IL-1β	GCAACTGTTCCTGAACTCAACT	ATCTTTTGGGGTCCGTCAACT
Mouse-IL-6	TAGTCCTTCCTACCCCAATTTCC	TTGGTCCTTAGCCACTCCTTC
Mouse-β-actin	GGCTGTATTCCCCTCCATCG	CCAGTTGGTAACAATGCCATGT
